# Large Extracervical Posterior Pedunculated Leiomyoma of the Uterus With the Only Symptom of Chronic Low Back Pain From Radiculopathy: A Case Report

**DOI:** 10.7759/cureus.48324

**Published:** 2023-11-05

**Authors:** Anna Thanasa, Efthymia Thanasa, Emmanouil M Xydias, Apostolos C Ziogas, Evangelos Kamaretsos, Ioannis Paraoulakis, Vasiliki Grapsidi, Ektoras-Evangelos Gerokostas, Ioannis Rafail Antoniou, Ioannis Thanasas

**Affiliations:** 1 Department of Health Sciences, Aristotle University of Thessaloniki, Thessaloniki, GRC; 2 Department of Obstetrics and Gynecology, Embryoclinic IVF Unit, Thessaloniki, GRC; 3 Department of Obstetrics and Gynecology, University of Thessaly, Larissa, GRC; 4 Department of Obstetrics and Gynecology, General Hospital of Trikala, Trikala, GRC

**Keywords:** extracervical leiomyoma, low back pain, radiculopathy, ultrasound, magnetic resonance imaging, surgical treatment, case report

## Abstract

Large extra cervical-type posterior subserosal leiomyomas originating from the cervix are extremely rare. Our case concerns the surgical treatment of a large posterior pedunculated subserosal extracervical leiomyoma of the uterus with extension to the retroperitoneal space, which was associated with chronic low back pain. A 45-year-old patient, without menstrual disorders and with a medical history of chronic low back pain with sciatica, was referred for gynecological evaluation. The gynecological examination revealed the presence of a large pelvic mass, which occupied the pouch of Douglas. Preoperative imaging confirmed the presence of a solid pelvic mass, but its origin could not be clarified. Neither transvaginal ultrasound nor MRI could establish the diagnosis of extracervical leiomyoma of the uterus. Based on the clinical and imaging findings, surgical management of the patient was decided with laparotomy. Intraoperatively, a large extracervical pedunculated leiomyoma was found, which originated from the posterior wall of the cervix and extended into the retroperitoneal space. An abdominal total hysterectomy and bilateral salpingo-oophorectomy were performed. The procedure had significant surgical difficulties. The postoperative course was uneventful. Three months after surgery, the patient reported relief of symptoms. This paper highlights a brief review of cervical leiomyomas, highlighting the important difficulties regarding the preoperative diagnosis and surgical management of these patients.

## Introduction

Fibroids or leiomyomas or myomas are common benign neoplasms of the uterus. Uterine leiomyomas originate from smooth muscle cells of the myometrium, whose growth depends mainly on the levels of endogenous estrogens. They usually occur in premenopausal women (<50 years) and are more common in the black race as compared to the white [1,2]. Leiomyomas are classified based on the International Federation of Gynecology and Obstetrics (FIGO) staging as cervical or extracervical leiomyomas [3].

Large cervical fibroids (>10cm) are even rarer [4]. Cervical leiomyomas are rare and estimated to account for less than 5% of all uterine fibroids [5]. Based on their anatomical location, they are divided into intracervical type, which refers to fibroids that grow inside the cervical lumen, and extracervical type, which refers to intramural or subserosal fibroids with or without pedicle, which is characterized by the growth of the tumor outside the cervix. Extracervical leiomyomas, which usually originate from the supra-vaginal part of the cervix, depending on their location of origin, can be further classified as anterior, posterior, and lateral. Cervical leiomyomas usually lack a pedicle and may develop within the broad ligament or extend into the retroperitoneal space [6].

This case report emphasizes the significant surgical difficulties that may arise in the management of extracervical leiomyomas of the uterus with extension to the retroperitoneal space, due to the increased risk of intraoperative bleeding and injury to adjacent organs. At the same time, it is pointed out that, despite their rarity and the considerable difficulties involved in preoperative diagnosis, extracervical leiomyomas of the uterus, particularly those growing in the retroperitoneal space, should be included in the differential diagnosis of chronic low back pain from radiculopathy in order to ensure health and quality of life for these patients.

## Case presentation

Our case concerns a patient aged 45 years old who was referred from the orthopedic clinic for examination at the gynecology outpatient clinic of the General Hospital of Trikala. The reason for the referral of the patient for gynecological evaluation was the existence of a possible pelvic mass, as it was depicted during the imaging of the lumbar spine with an MRI. An MRI revealed no evidence of bone damage or intervertebral disc disease in the lumbar spine. The patient complained of symptoms of persistent low back pain with accompanying radicular pain in the left leg for the last two years. As reported by the patient herself, the symptoms did not completely resolve after the analgesic medication that she occasionally received. The patient had a regular menstrual cycle and had given birth to three children by vaginal delivery. No previous surgeries were mentioned in the personal medical history. Medical disorders such as hypothyroidism, arterial hypertension, and anxiety disorders were reported and well-regulated with appropriate medication.

**Figure 1:**
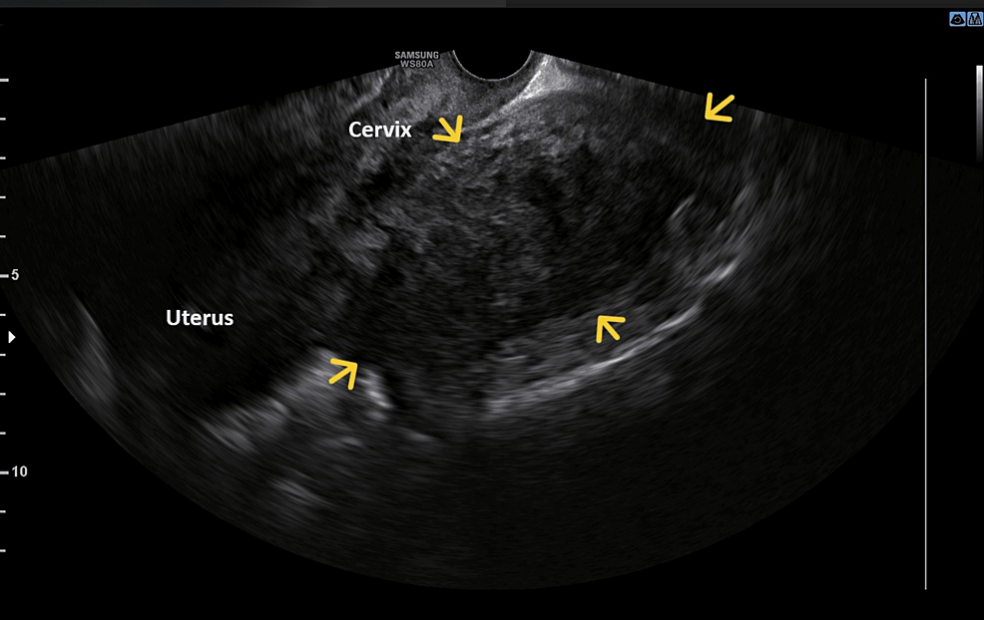
Transvaginal ultrasound of the posterior pedunculated extracervical leiomyoma of the uterus The demarcation (yellow arrows) of a large echogenic mass with solid elements, which occupies the pouch of Douglas, is indicated

During the gynecological examination, a large palpable pelvic mass was found, but it could not be determined whether it was a uterine mass or an adnexal mass. The cervix of the uterus was observed to have smooth, squamous epithelium but appeared to be displaced. Transvaginal ultrasound revealed a large solid echogenic mass (maximum diameter of about 11 cm) occupying the pouch of Douglas, but it was not possible to clarify the exact origin of the pelvic mass (Figure 1).

**Figure 2:**
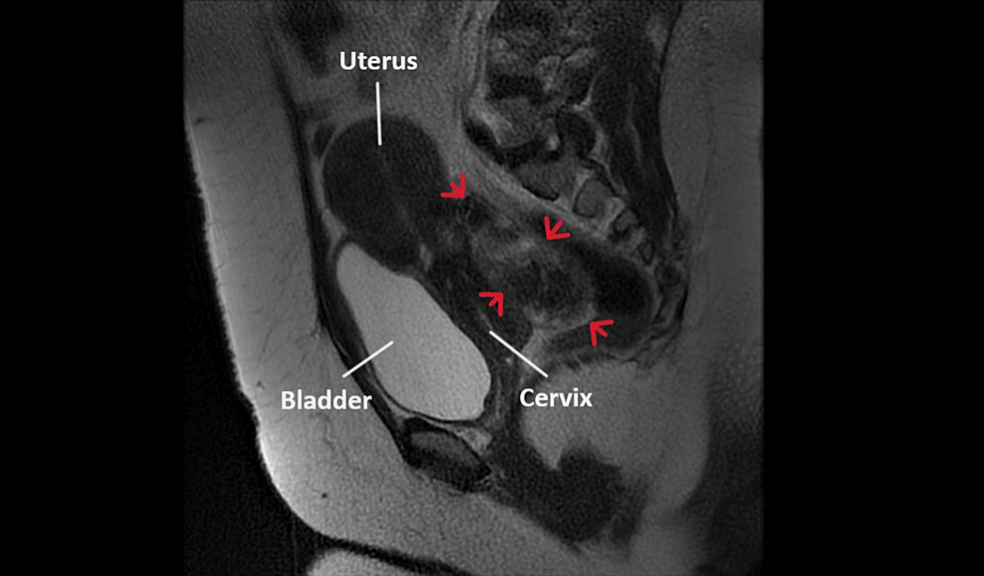
MRI of a posterior pedunculated extracervical leiomyoma of the uterus The pelvic space-occupying lesion depicted with red arrows represents an extracervical-type posterior pedunculated leiomyoma of the uterus, whose extension into the retroperitoneal space cannot be clarified

MRI revealed a large oval-shaped mass originating from the right lateral side of the uterine corpus of dimensions 10.88x8.7x6.3 cm, with a heterogeneous enhancement well-defined by a thin wall that extended into the pouch of Douglas (Figure 2).

The left ovary was normal in size and consistency, while the right ovary was not depicted in the current imaging. The findings from the MRI were more consistent with a subserosal leiomyoma of the uterine corpus, while a preoperative diagnosis of an adnexal mass was less likely. The patient's blood tests were hematocrit 40.4%, hemoglobin 13.1 g/dl, WBC 5,810/ml, neutrophil 59%, platelet 218x103/ml, international normalized ratio 1.06, fibrinogen 282 mg/dl, urea 27 mg/dl, and creatinine 0.75 mg/dl. A pap smear test was negative for malignancy. The tumor markers (CEA, Ca125, Ca15-3, Ca19-9) were within the normal range.

**Figure 3:**
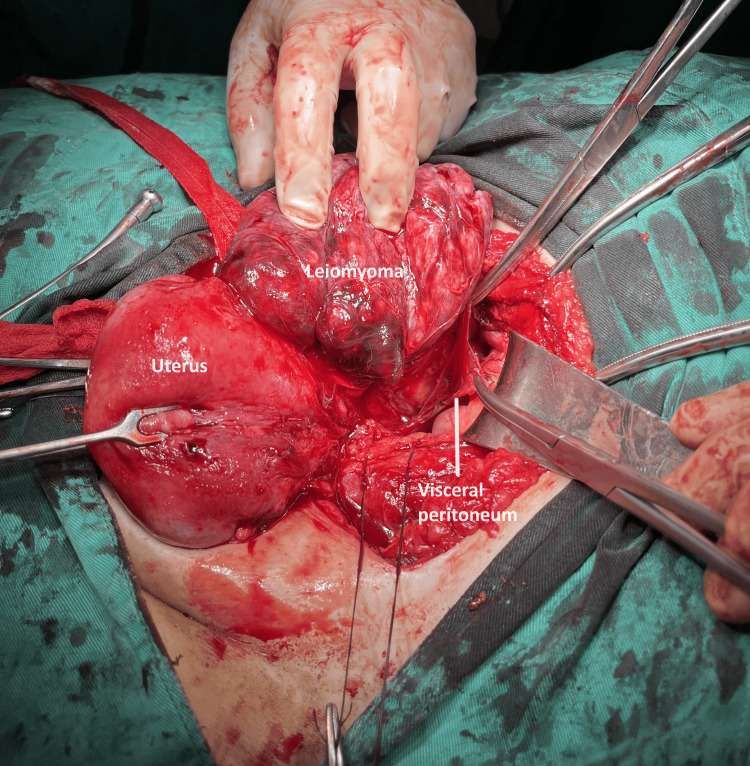
Intraoperative imaging of posterior pedunculated extracervical leiomyoma of the uterus The extension of the tumor into the retroperitoneal space is evident

Based on the clinical and imaging findings, it was considered that there is a strong possibility that the symptom of persistent low back pain with concomitant sciatica was due to compression on the lumbar nerve roots from the large pelvic mass that occupied the pouch of Douglas. After consulting the patient about the available treatment options, it was decided to perform a diagnostic laparotomy, with the possible necessity of performing an abdominal total hysterectomy. Intraoperatively, a large extracervical leiomyoma was found, which, through a wide-based pedicle, emerged from the posterior wall of the cervix and which, with retroperitoneal extension, occupied the entire pouch of Douglas (Figure 3).

**Figure 4:**
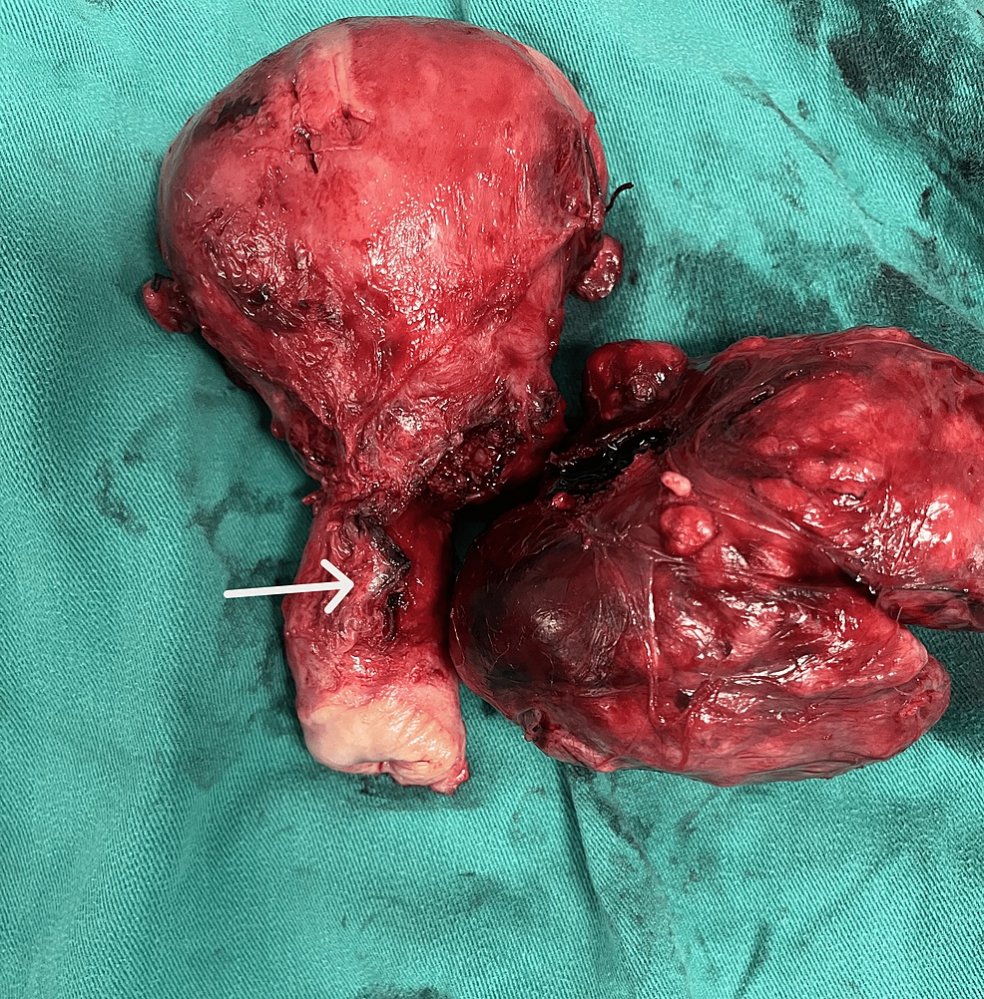
Imaging of surgical specimen after resection of cervical leiomyoma and performing of abdominal total hysterectomy with bilateral adnexectomy The extracervical origin of the leiomyoma and the presence of the tumor's attachment site (white arrow) from the posterior supravaginal wall of the cervix are evident

A marked displacement of the anatomical structures in the pelvic area was noted. Careful dissection of the distal ureters up to their entry into the bladder was performed while attempting to preserve their vascularization and avoid injury to the iliac vessels and their branches passing through the anatomical area. In this way, the separation of the cervical leiomyoma from the adjacent structures was safely completed, and it was possible to extract it with the uterus and adnexa (Figure 4).

**Figure 5:**
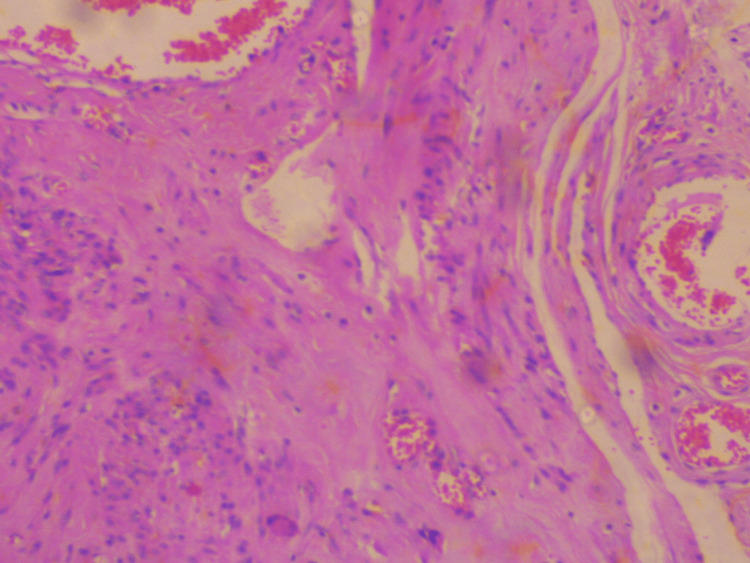
Histological image of extracervical leiomyoma of the uterus Moderate cellularity, with the absence of mitoses, necrosis, and atypia, is depicted

**Figure 6:**
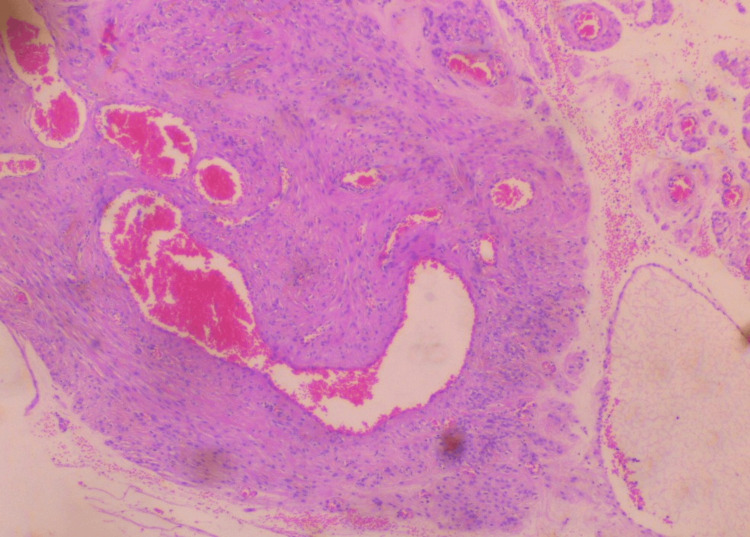
Histological image of extracervical leiomyoma of the uterus Vitrification of the stroma and vascular congestion, probably due to compression, is depicted

Surgery was completed without significant blood loss. Histological examination of the surgical specimen confirmed the diagnosis of extracervical leiomyoma of the uterus. Microscopic examination of the tumor revealed mild to moderate cellularity, with the absence of mitoses, necrosis, and atypia. In addition, vitrification of the stroma and vascular congestion, probably due to compression, were found in some places (Figures 5-6).

After a smooth postoperative course, the patient was discharged from the clinic on the fourth postoperative day, with consultation for re-examination at the gynecological outpatient clinic. Three months postoperatively, the patient reported complete relief of symptoms of chronic lower back pain with accompanying radicular pain.

## Discussion

The preoperative diagnosis and surgical management of patients with extracervical leiomyomas are estimated to be much more challenging compared to the management of those patients in whom fibroids are located in the uterine corpus [7]. Urinary retention and frequent urination are the main symptoms in cases where the extracervical leiomyoma is located at the anterior cervical wall, while constipation and painful defecation characterize cases with the presence of a large mass originating from the posterior cervical wall. Additionally, abnormal uterine bleeding, chronic pelvic pain, dysmenorrhea, and dyspareunia are typical symptoms that usually characterize cervical leiomyomas [4,8]. Our patient did not complain of any symptoms related to menstruation or rectal compression from the tumor. Remarkably, in our patient, the only clinical manifestation was chronic low back pain due to radiculopathy. The persistent low back pain accompanied by sciatica is most likely due to the presence of a large exophytic posterior cervical leiomyoma, which occupied the pouch of Douglas and caused compression or irritation of one or more lumbar spine nerve roots. This resulted in low back pain radiating down the leg along the affected nerve root. This is supported by the fact that the MRI of the lumbar spine did not show any bone damage. No herniated disc with nerve root compression, no degenerative disc disease of the lumbar spine, no narrowing of the intervertebral discs, or spinal stenosis were found. Furthermore, her medical history did not indicate any previous surgical treatment on the lumbar spine that could, through the development of scar tissue, explain the irritation or inflammation of the nerve roots originating from the lumbar spine.

The diagnostic value of ultrasonography and MRI, or a combination of the two, is considered to be important in the preoperative diagnostic evaluation of cervical leiomyomas of the uterus. Recently, in 2021, Ferrari et al. showed that the diagnosis of cervical leiomyoma can be made by ultrasound in about half of the patients (48.6%) and by MRI in 39% of cases [9]. In a previous study, Sinha et al. showed that, using diagnostic ultrasonography, only one case of misdiagnosis was found in a total of 89 patients with cervical leiomyomas [10]. Nevertheless, in our case, neither transvaginal ultrasound nor MRI could determine a preoperative diagnosis of extracervical leiomyoma of the uterus. On transvaginal ultrasound, there were findings more consistent with a large subserosal fibroid of the uterine corpus, without a diagnosis of the extracervical origin of the leiomyoma being established with certainty. Furthermore, pelvic MRI revealed a large pelvic mass more compatible with a subserosal uterine fibroid, while the findings were less consistent with the presence of a dermoid cyst from the right ovary. In our patient, the diagnosis of extracervical leiomyoma of the uterus was made intraoperatively.

The treatment of cervical leiomyomas of the uterus is mainly surgical. Hysterectomy with laparotomy or laparoscopy is recommended as the primary treatment option for older women. Myomectomy and trachelectomy should be offered to patients who wish to preserve fertility and achieve a future pregnancy. Myomectomy is advisable to be limited to fibroids smaller than 10 cm. In addition to the increased risk of intraoperative bleeding, the risk of recurrence and the need to perform a hysterectomy should be included among the common complications associated with cervical leiomyoma resection [11]. Additionally, it is considered necessary to inform patients regarding the possible complications and the possible poor outcome of future pregnancies while undergoing cervical myomectomy [12]. Recently, in 2022, Zhang et al. showed that for surgical resection of posterior cervical leiomyoma of the uterus, the laparoscopic approach is safe [13]. In our patient, abdominal total hysterectomy with adnexectomy was considered the treatment of choice. Our patient preoperatively consented to the recommended surgery, as she had completed her family planning and did not wish to preserve fertility.

Regardless of the type of surgery (hysterectomy, myomectomy, or trachelectomy) chosen to treat cervical leiomyoma, the operation is expected to be difficult. A well-organized preoperative evaluation of the patient and thorough knowledge by the surgical team of the pelvic anatomy, which is usually deformed by the cervical leiomyoma, especially when it extends between the broad ligaments or in the retroperitoneal space, are essential for a good outcome of the surgery. In addition, the surgical skills of the team of gynecologists and a well-organized operating room should be included in the requirements for the successful and safe treatment of cervical leiomyomas of the uterus. Significant intraoperative blood loss due to increased cervical blood perfusion, inadequate surgical plan, and increased risk of bladder, rectal, or ureteral injury are complications that often occur in the surgical treatment of cervical leiomyomas [7,14]. Ureteral injury can be prevented by preoperative bilateral placement of ureteral stents, which allow safe intraoperative ureter dissection and avoid injury from surgical maneuvers while attempting to resect the mass [15]. In addition, preoperative treatment with a GnRH agonist, intraoperative intramyometrial injection of vasopressin, temporary uterine artery occlusion, permanent bilateral ligation of the uterine arteries, and the use of an internal iliac artery balloon occlusion catheter are useful to include in procedures required to prevent intraoperative hemorrhage in the treatment of cervical leiomyomas [16,17].

In our patient, intraoperatively, a remarkable extension of the extracervical posterior pedunculated subserosal leiomyoma (>10cm) in the retroperitoneal space and a significant degree of displacement of adjacent anatomical structures, including the ureters, were detected. This condition required careful dissection of the ureters from the adjacent tissues, particularly the right ureter, which had greater misplacement, in order to preserve sufficient blood supply and avoid injury. Furthermore, avoiding injury to the great blood vessels that pass through the anatomical area was of primary importance in order to achieve a safe dissection of the cervical leiomyoma and to exclude it along with the uterus. In our patient, no ureteral stents were placed preoperatively because there was no such possibility in our hospital. This fact further increased the degree of difficulty of the surgery.

## Conclusions

Large cervical leiomyomas are rare. Early diagnosis of chronic low back pain due to radiculopathy-associated extracervical leiomyoma is critical for the optimal surgical treatment to be applied immediately. The preoperative diagnosis and surgical treatment of large extracervical posterior cervical leiomyomas of the uterus is a challenge today in routine clinical practice. Hysterectomy, myomectomy, or trachelectomy, depending on the age of the patient and her desire or not to preserve fertility, in well-organized medical centers by experienced surgeons, is considered essential for the treatment of symptomatic cervical leiomyomas of the uterus in order to ensure the health and quality of life of these patients.
